# Cerebrospinal fluid levels of the neurotrophic factor neuroleukin are increased in early Alzheimer’s disease, but not in cerebral amyloid angiopathy

**DOI:** 10.1186/s13195-021-00899-0

**Published:** 2021-09-24

**Authors:** Anna M. De Kort, H. Bea Kuiperij, Daniel Alcolea, Iris Kersten, Alexandra A. M. Versleijen, Steven M. Greenberg, Erik Stoops, Floris H. B. M. Schreuder, Catharina J. M. Klijn, Alberto Lleó, Jurgen A. H. R. Claassen, Marcel M. Verbeek

**Affiliations:** 1 Department of Neurology, Radboud University Medical Center, Donders Institute for Brain, Cognition and Behaviour, Radboud Alzheimer Centre, P.O. Box 9101, 6500 HB Nijmegen, The Netherlands; 2 Sant Pau Memory Unit, Department of Neurology, Hospital de la Santa Creu i Sant Pau, Biomedical Research Institute Sant Pau, Universitat Autònoma de Barcelona, Barcelona, Spain; 3 Center of Biomedical Investigation Network for Neurodegenerative Diseases (CIBERNED), Madrid, Spain; 4 Department of Laboratory Medicine, Radboud University Medical Center, Nijmegen, The Netherlands; 5 Department of Neurology, Massachusetts General Hospital, Harvard Medical School, Boston, MA USA; 6 ADx NeuroSciences, Ghent, Belgium; 7 Department of Geriatrics, Radboud University Medical Center, Donders Institute for Brain, Cognition and Behaviour, Radboud Alzheimer Centre, Nijmegen, The Netherlands

**Keywords:** Cerebrospinal fluid, Biomarkers, Alzheimer’s disease, Amyloid, Cerebral amyloid angiopathy, Neuroleukin, Neuro-inflammation

## Abstract

**Background:**

Neuroleukin (NLK) is a protein with neurotrophic properties and is present in a proportion of senile plaques and amyloid laden vessels. It has been suggested that NLK is part of a neuroprotective response to amyloid β-induced cell death. The aim of our study was to investigate the value of cerebrospinal fluid (CSF) NLK levels as a biomarker of vascular amyloid deposition in patients with cerebral amyloid angiopathy (CAA) and in patients with amnestic mild cognitive impairment (aMCI) and Alzheimer’s disease (AD).

**Methods:**

CSF NLK levels were quantified by ELISA in CAA patients (*n* = 25) and controls (*n* = 27) and in two independent samples of aMCI patients, AD patients, and controls: (1) From the Radboud University Medical Center (Nijmegen), we included *n* = 19 aMCI patients, *n* = 40 AD patients, and *n* = 32 controls. (2) From the Hospital of Sant Pau (Barcelona), we included *n* = 33 aMCI patients, *n* = 17 AD patients, and *n* = 50 controls.

**Results:**

CSF NLK levels were similar in CAA patients and controls (*p* = 0.95). However, we found an elevated CSF concentration of NLK in aMCI (*p* < 0.0001) and AD patients (*p* < 0.0001) compared to controls in both samples sets. In addition, we found a correlation of CSF NLK with CSF YKL-40 (age-adjusted-spearman-rank-coefficient = 0.82, *p* < 0.0001) in aMCI/AD patients, a well-known glial marker of neuro-inflammation.

**Conclusions:**

We found that CSF NLK levels are elevated in aMCI and AD patients compared to controls, but are not increased in CAA patients. CSF NLK levels may be related to an increased neuroinflammatory state in early stages of AD, given its association with YKL-40.

## Introduction

Alzheimer’s disease (AD) is the leading cause of dementia worldwide. It is neuropathologically characterized by the intracellular accumulation of hyperphosphorylated tau protein in neurofibrillary tangles and by the extracellular accumulation of amyloid-beta protein (Aβ). In AD, Aβ is found in parenchymal senile plaques, and in many cases also in the cerebral vasculature, referred to as cerebral amyloid angiopathy (CAA) [1]. The clinical diagnosis of AD is based on a gradual decline of cognition, most commonly with an impairment in encoding and recall of recently learned information as well as evidence of cognitive dysfunction in at least one other cognitive domain [2]. Mild cognitive impairment (MCI) refers to an early, but abnormal state of cognitive impairment defined by memory complaints, objective memory impairment, essentially preserved general cognitive function, largely intact functional activities, but not demented [3]. Cerebrospinal fluid (CSF) and neuroimaging biomarkers can be used to support the diagnosis [4] and have been proposed to define a prodromal stage of AD based on the presence of MCI and evidence for amyloid deposition (A), tau accumulation (T), and neurodegeneration (N) [4]. Beyond their supporting role in the diagnosis, biomarkers may contribute to predicting prognosis and may facilitate monitoring of disease progression and treatment response. Furthermore, novel biomarkers may advance our comprehension of the different aspects of AD pathophysiology next to tau and amyloid pathology.

CAA is the most prevalent co-pathology of AD, with almost 80% of the patients having some degree of CAA [5], likely because Aβ deposition is a shared feature of senile plaques and CAA [6]. Vascular Aβ depositions can also occur in the absence of AD and is recognized as a designated type of cerebral small vessel disease, referred to as sporadic CAA. Sporadic CAA is associated with cerebral (micro) hemorrhages and can also result in a decline in cognitive function [5]. Sporadic CAA is diagnosed during life using the MRI-based modified Boston criteria [7], which rely on the hemorrhagic manifestations of CAA. However, these are indirect signs of relatively late-stage manifestations of CAA and they do not provide definite proof of the disease.

Neuroleukin (NLK) may serve as biomarker for CAA or AD. In an immunohistochemical study in AD patients, NLK expression was found in senile plaques and in Aβ-laden vessels [8]. In addition, messenger RNA levels of NLK were increased in cultured human brain pericytes after exposure to Aβ40 [8], which may represent an attempt to survive Aβ-induced cell death. Furthermore, it was shown that NLK can serve as a neurotrophic growth factor [9] and may support survival of sensory and spinal neurons and stimulate axonal growth [10–13].

CSF YKL-40 (also known as chitinase-3 like-protein 1 or cartilage glycoprotein-39) is an established neuroinflammatory marker [14]. YKL-40 is mainly expressed by astrocytes, but also by microglia, often in close vicinity to senile plaques and neurofibrillary tangles [15–17]. Increased CSF YKL-40 levels have been reported in cognitively healthy APOE-ε4 carriers, amnestic mild cognitive impairment (aMCI), and AD patients [14] and also in frontotemporal dementia [18] and amyotrophic lateral sclerosis [19].

The aim of our study was to examine CSF NLK levels in patients with CAA and in two independent samples of patients with aMCI or AD diagnosed according to clinical criteria. We also studied the association of CSF NLK in aMCI and AD patients with a positive biomarker status A+T+(N+), and we performed an exploratory study on the association of CSF NLK with CSF YKL-40, and the known Alzheimer CSF biomarkers total tau, phosphorylated tau, and Aβ42.

## Methods

### Participants

#### Sporadic CAA patients and controls

We included 14 patients with sporadic CAA from the Massachusetts General Hospital (MGH), Boston, USA, and 11 patients with sporadic CAA and 27 controls from the Radboud University Medical Center, Nijmegen, the Netherlands (RUMC). The inclusion criteria for the CAA patients were a diagnosis of probable or definite CAA according to the modified Boston criteria [7]. MMSE scores were available for a subset (*n* = 15) of these patients (Table 1). Controls underwent a lumbar puncture as part of the diagnostic workup of neurologic symptoms or to exclude central nervous system involvement of a systemic disease at the RUMC. They neither had the suspected neurological disease nor a neurodegenerative disease, known cognitive impairment, sepsis, a recent stroke (< 6 months), or a malignancy in the central nervous system.

**Table 1:** Characteristics of the sporadic CAA patients and controls

	CAA (*n* = 25)	Controls (*n* = 27)	*p*-value
Age (years)	66 ± 10	63 ± 10	*p* = 0.32^a^
Sex, M/F (*n*)	13/12	12/15	*p* = 0.59^b^
MMSE score	28 [26–29]^d^	N.A.	*-*
TP (mg/ml)	0.91 [0.85–0.96]	0.83 [0.78–1.03]	*p* = 0.12^c^
Aβ40 (pg/ml)	3745 [2200–4694]	6312 [4888–7381]	*p* < 0.0001^c^
NLK (ng/ml)	3.39 [2.94–4.15]	3.31 [2.41–5.40]	*p =* 0.95^c^

Values are medians and [IQR] except for age (mean ± SD) and sex (*n*). TP, NLK, and Aβ40 were measured in CSF

*Abbreviations: Aβ40* amyloid beta-40, *CAA* cerebral amyloid angiopathy, *F* female, *IQR* interquartile range, *M* male, *MMSE* Mini-Mental State Examination, *N.A.* not available, *NLK* neuroleukin, *SD* standard deviation, *TP* total protein

^a^Student’s *t*-test

^b^Chi-square test

^c^Mann-Whitney test

^d^*n* = 15 patients

#### aMCI and AD patients and controls

We included two independent samples of patients with amnestic mild cognitive impairment (aMCI), patients with AD, and controls: we performed an initial analysis including participants from the RUMC (the “Nijmegen aMCI/AD patients and controls”) and a confirmatory analysis in participants from the Memory Unit at Hospital Sant Pau, Barcelona (the “Barcelona aMCI/AD patients and controls”).

##### Nijmegen aMCI/AD patients and controls

We included 19 patients with aMCI, 40 patients with probable AD, and 32 controls (different than the above-mentioned *n* = 27), all from the RUMC. The inclusion criteria were either a clinical diagnosis of aMCI according to the Petersen criteria [3] at the moment of the lumbar puncture followed by a diagnosis of probable AD according to NINCDS-ADRDA criteria [20] in a later stage, or a diagnosis of probable AD according to the same criteria. Diagnoses were established during multidisciplinary memory clinic meetings. We used predefined local cutoff values of CSF analysis to stratify the patients and controls for their “ATN status” (Aβ42 (A+), < 500 pg/ml; phosphorylated tau (T+), > 85 pg/ml; total tau (N+), > 350 pg/ml) [21]. Mini Mental State Examination (MMSE) scores [22] were available for all aMCI and AD patients (Table 2). These patients were compared with 32 controls from RUMC, fulfilling the criteria as mentioned above (“ Sporadic CAA patients and controls”).

**Table 2:** Characteristics of Nijmegen aMCI/AD patients and controls

	aMCI (*n* = 19)	AD (*n* = 40)	Controls (*n* = 32)	*p*-value
Age (years)	72 [70–78]	74 [65–77]	61 [56–67]	*p* < 0.0001^a^
Sex, M/F (*n*)	7/12	11/29	15/17	*p* = 0.24^b^
MMSE score	26 [23–27]	20 [17–24]	N.A.	*p* < 0.0001^a^
APOE genotype	ε4+: *n* = 10 ε4−: *n* = 3 N.A.: *n* = 6	ε4+: *n* = 0 ε4−: *n* = 8 N.A.: *n* = 2	N.A.	*p* = 0.58^b^
TP (mg/ml)	0.87 [0.79–0.93]	0.85 [0.76–0.96]	0.91 [0.79–.02]	*p* = 0.51^a^
T-tau (pg/ml)	504 [419–691]	627 [321–956]	267 [179–386]	*p* < 0.0001^a^
P-tau (pg/ml)	91.0 [71.0–114.0]	97.5 [79–129]	47.5 [32.3–56.8]	*p* < 0.0001^a^
Aβ42 (pg/ml)	459 [404-516]	468 [389–564]	864 [664–1142]	*p* < 0.0001^a^
NLK (ng/ml)	4.66 ± 1.40	4.84 ± 1.58	3.07 ± 1.2	*p* < 0.0001^d^
GCA score	*n* = 7; 2 [2–2]	*n* = 28; 1.5 [1–2]	*n* = 4; 0.5 [0–1]	*p* = 0.006^c^
Mean MTA	*n* = 5; 1.5 [1–2.25]	*n* = 28; 1 [1–2]	*n* = 2; 0 [0–0]	*p* = 0.125^c^

Medians and [IQR] are reported for all characteristics and markers, except for NLK (mean ± SD) and sex (*n*). T-tau, P-tau, Aβ42, and NLK were measured in CSF

*Abbreviations: AD* Alzheimer’s disease, *Aβ42* amyloid beta-42, *APOE* Apolipoprotein E, *aMCI* amnestic mild cognitive impairment, *F* female, *GCA* global cortical atrophy, *IQR* interquartile range, *M* male, *MMSE* Mini-Mental State Examination, *MTA* medial temporal lobe atrophy, *N.A.* not available, *NLK* neuroleukin, *P-tau* phosphorylated tau, *SD* standard deviation, *TP* total protein, *T-tau* total tau

^a^Kruskal-Wallis test

^b^Chi-square test

^c^Fisher’s exact test

^d^ANOVA

##### Barcelona aMCI/patients and controls

For validation of our findings, we included 33 patients with aMCI, 17 patients with probable AD, and 50 cognitively normal controls from the SPIN cohort (Memory Unit at Hospital Sant Pau, Barcelona). Inclusion criteria were a diagnosis of aMCI according to the Petersen criteria [3] or probable AD according to the NINCDS-ADRDA criteria [20]. Controls were spouses or children of patients, and inclusion criteria were the absence of memory complaints or significant impairment in other domains or daily living activities, a clinical dementia rating global score of 0, an MMSE score of > 27, and a Free and Cued Selective Reminding Test (FCSRT) total immediate score within normal range for age and education [23]. Mini Mental State Examination (MMSE) scores [22] were available for all aMCI, AD patients, and controls (Table 2).

We used previously published local cutoff values to stratify the patients and controls for their “ATN status” (Aβ42 (A+), < 916 pg/ml; phosphorylated tau (T+), > 63 pg/ml; total tau (N+), > 456 pg/ml) [24].

### CSF analysis

All participants in this study underwent a lumbar puncture according to state-of-the-art local protocols. At all locations, the CSF was collected in polypropylene tubes, centrifuged, aliquoted, and stored in polypropylene tubes at − 80 °C. For all CSF analyses, the technician who performed the analysis was blinded for the clinical diagnoses, and patient and controls samples were randomly analyzed to overcome bias.

NLK was quantified in CSF using the human glucose-6-phosphate isomerase ELISA kit (cat no. Ab171575; Abcam, Cambridge, UK) according to the manufacturers’ instructions [25]. A standard curve ranging from 156 to 10,000 pg/ml was constructed using serial two-fold dilutions. CSF samples were diluted six times prior to analysis.

Total protein levels in CSF were determined using Pierce^TM^ BCA protein assay kit (Thermo Fisher Scientific, Waltham, MA, USA). In all NLK and total protein CSF analyses, five quality controls were included on each plate in each test run to correct for any inconsistencies between plates. These controls consisted of pooled CSF samples that were stored in aliquots at −80 °C. For each analysis, a fresh aliquot was used.

Aβ42, tau phosphorylated at threonine 181 (p-tau), and total tau (t-tau) levels in CSF of Nijmegen aMCI/AD patients and controls were analyzed using Innotest ELISAs (Fujirebio, Gent, Belgium). Test characteristics of these assays have been described previously [26]. In Barcelona aMCI/AD patients and their controls, these biomarkers were analyzed using automated versions of the same assays using a Lumipulse apparatus (Fujirebio, Gent, Belgium).

Aβ40 levels in CSF of CAA patients and their controls were analyzed using an ELISA (Euroimmun, Lübeck, Germany). *APOE* genotyping was performed as described earlier [27].

CSF YKL-40 measurements were available for a subset (*n* = 29 controls, *n* = 13 aMCI, *n* = 5 AD patients) of the Barcelona patients and controls. These were analyzed as described earlier (MicroVue^TM,^ Quidel, San Diego, CA) [28].

### MRI acquisition and analysis

MRIs were obtained in a clinical setting, and field strength was either 1.5 or 3.0 T. MRI analysis included axial and coronal T1-weighted and T2-weighted sequences. MRI images were reviewed blinded to all clinical and CSF data and scored for medial temporal lobe atrophy (MTA) [29] and global cortical atrophy (GCA) [30]. The MTA scale assesses the width of the choroidal fissure and the temporal horn, and the height of the hippocampal formation on a 5-grade rating scale [29]. Both hemispheres were scored, and the mean score of the left and right hemisphere was used. The GCA score is the mean score for cortical atrophy throughout the entire cerebrum and was rated by a 4-point scale [30]. Each MRI was rated independently by two trained raters (AK and LR), and there was a good agreement between the two raters: a weighted Cohen’s *κ* of 0.69 for GCA and 0.73 for MTA. In case of a disagreement, consensus was reached by consultation of a skilled vascular geriatrician (JC).

### Data analysis

We used the software programs IBM SPSS statistics for Windows, version 25.0 (Armonk, NY: IBM Corp) and GraphPad Prism 5.03 (La Jolla, CA). If parameters had a Gaussian distribution, parameters were depicted as mean ± standard deviation and group differences were analyzed with a Student’s *t*-test or an ANOVA. Otherwise, parameters were stated as medians with interquartile ranges and differences were analyzed with a Mann-Whitney *U* test or a Kruskal-Wallis test. The Shapiro-Wilk test was used to analyze the normality of the data. Sex frequency was analyzed by a chi-square test. GCA and MTA were compared using Fisher’s exact test. When comparing group differences of NLK levels, we adjusted for age and sex by performing multiple regression analysis with patient group, age and sex as independent variables. In a second model, we adjusted for age, sex, and CSF total protein levels. To explore potential specific relationships between NLK and Alzheimer pathology, we performed a subgroup analysis of aMCI/AD patients who were “AD biomarker positive” (i.e., A+T+(N+)) compared with “AD biomarker-negative” (i.e., A−T−(N−)) controls. We pooled aMCI and AD biomarker positive individuals to increase the power of this analysis. To determine the diagnostic accuracy of NLK for the distinction between aMCI patients and controls, and between AD patients and controls, we determined the area under the curve (AUC) using a receiver operating characteristic curve (ROC) with 95% confidence interval (CI). Spearman rank correlation (*r*_SP_) was used to evaluate correlation between age, total protein, and MMSE score with NLK. We used partial correlation with age as a covariate to adjust for age when investigating the association between t-tau, p-tau, Aβ42, YKL-40, GCA, MTA, and NLK. To further explore the association of NLK with YKL-40, we also performed this analysis in the controls and the MCI/AD patients separately.

### Ethical statement

Lumbar punctures and vena punctures were performed after informed consent from the patients themselves or from the patients’ legal representatives. The CSF of AD patients, controls, and the majority of the CAA patients from the RUMC (*n* = 7) originated from a clinical diagnostic work-up. Four CAA patients underwent a lumbar puncture in the context of a cohort study on biomarkers for CAA (CAVIA; no. 733050202). This study was approved by the Medical Ethics Committee Arnhem-Nijmegen (file nos. 2016-3011 and 2014-1401), Ethical Committee of IIB-Sant Pau (16/2013), and Partners Human Research Committee of Boston (study ID PHRC #2006P000664).

## Results

### NLK assay for quantification in CSF

The lower limit of detection (LLOD) of the NLK ELISA was 61 pg/ml. All CSF measures were above the LLOD. The mean intra-assay coefficient of variation (CV) for CSF samples (duplicate measures) was 3.6 ± 3.6% and the mean inter-assay CV was 4.9 ± 2.9%. The variation between the ELISA plates, based on quality control measurements, ranged from a factor 0.89 to 1.08, for which the CSF measures were corrected accordingly, by recalculating the results using the results of one plate as a reference.

### CSF NLK levels in sporadic CAA patients and controls

**Fig. 1:**
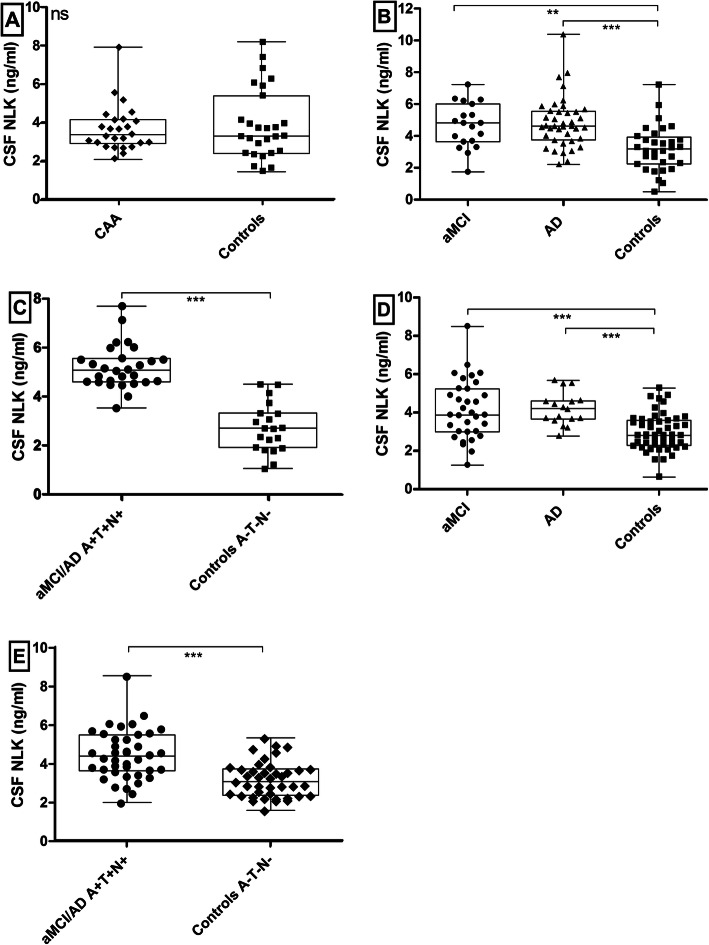
CSF NLK levels in the sporadic CAA patients and controls (**A**) and in the aMCI/AD patients and controls (**B**–**E**), with and without stratification for AT(N) status. Box and scatterplots in all panels (depicting median, interquartile range and range). **A** NLK levels in CAA patients and controls. The NLK levels were similar (*p* = 0.95). **B** CSF NLK levels in aMCI patients, AD patients, and controls from Nijmegen. These levels were increased in aMCI (*p* = 0.001) and AD patients (*p* < 0.0001) compared to controls. **C** NLK levels in A+T+(N+) aMCI/AD patients and A−T−(N−) controls from Nijmegen. NLK levels were significantly higher in A+T+(N+) aMCI/AD patients (*p* < 0.0001). **D** CSF NLK levels in aMCI patients, AD patients, and controls from Barcelona. Levels were significantly higher in aMCI patients (*p* < 0.0001) and AD patients (*p* < 0.0001) compared to controls. **E** NLK levels in A+T+(N+) aMCI/AD patients and A−T−(N−) controls from Barcelona. NLK levels were significantly higher in A+T+(N+) aMCI/AD patients (*p* < 0.0001). Abbreviations: AD, Alzheimer’s disease; CAA, cerebral amyloid angiopathy; CSF, cerebrospinal fluid; aMCI, amnestic mild cognitive impairment; ns, non-significant. **p < 0.01, ***p < 0.001

Age and sex distribution of the CAA patients (age 66 ± 10 years, 52% male) and controls (age 63 ± 10 years, 44% male) was similar (Table 1). Patients were assigned to the following categories of certainty: definite CAA (autopsy confirmed, *n* = 2), probable CAA with supporting pathology (*n* = 6), and probable CAA (*n* = 17). CSF NLK levels, adjusted for age and sex, were similar (*p* = 0.40, see Table 1 and Fig. 1A) in CAA patients and controls. Additional adjustment for CSF total protein did not affect the results (*p* = 0.34).

We observed an increase in CSF NLK with increasing age (*r*_SP_ = 0.37, *p* = 0.014), but we found no correlation with total protein (*r*_SP_ = 0.008, *p* = 0.95). Furthermore, CSF NLK correlated with MMSE score (*r*_SP_ = −0.76, *p* = 0.001).

### CSF NLK levels in Nijmegen aMCI/AD patients and controls

Sex distribution was similar, whereas aMCI (median age 72 [70–78] years, 37% male) and AD patients (median age 74 [65–77] years, 28% male) were older (both *p* < 0.0001) than the control subjects (median age 61 [56–67] years, 47% male; Table 2). CSF NLK levels were, adjusted for age and sex, increased in aMCI patients compared to controls (*p* = 0.009; Table 2 and Fig. 1B) and in AD patients compared to controls (*p* < 0.0001). Additional adjustment for CSF total protein did not affect the results (*p* = 0.005 and *p* < 0.0001, respectively).

Furthermore, in *n* = 28 aMCI/AD patients with positive AD biomarkers (A+T+(N+)) the mean CSF NLK concentration (5.21 ± 1.08 ng/ml) was higher than in *n* = 20 controls with normal AD biomarker status (A−T−(N−); 2.72 ± 1.0 ng/ml; when adjusted for age and sex (*p* < 0.0001; Fig. 1C), and after additional adjustment for total protein (*p* < 0.0001).

CSF NLK significantly correlated with age (*r*_SP_ = 0.39, *p* < 0.0001) and with CSF total protein levels (*r*_SP_ = 0.22, *p* = 0.039).

### CSF NLK levels in Barcelona aMCI/AD patients and controls

Sex distribution was similar, whereas aMCI (mean age 70 ± 3 year, 30% male), and AD patients (mean age 67 ± 7 years, 47% male) were older (*p* < 0.0001 and *p* = 0.002) than the control subjects (mean age 63 ± 4 years, 50% male; Table 3). When adjusted for age and sex, there was a significant difference between aMCI patients and controls (*p* = 0.018; Table 3 and Fig. 1D), and between AD patients and controls (*p* = 0.008). Additional adjustment for CSF total protein yielded similar results (*p* = 0.013 and *p* = 0.005, respectively).

**Table 3:** Characteristics of the Barcelona patients and controls

	aMCI *n* = 33	AD *n* = 17	Controls *n* = 50	*p*-value
Age (years)	70 ± 3	67 ± 7	63 ± 4	*p* < 0.0001^a^
Sex, M/F (*n*)	10/23	8/9	25/25	*p* = 0.20^b^
MMSE score	26 [25–28]	20 [19–25]	29 [29–30]	*p* < 0.000^d^
APOE genotype	ε4+: n = 24 ε4−: n = 8 N.A.: *n* = 1	ε4+: *n* = 6 ε4−: n = 10 N.A.: *n* = 1	ε4+: *n* = 10 ε4−: *n* = 38 N.A.: *n* = 2	*p* < 0.0001^c^
TP (mg/ml)	0.78 [0.72–0.88]	0.79 [0.71–0.88]	0.78 [0.69–1.04]	*p* = 0.99^d^
T-tau (pg/ml)	647 [411–823]	*n* = 15; 742 [598–1163]	265.0 [227–305]	*p* < 0.0001^d^
P-tau (pg/ml)	101.3 [78.0–143.8]	114.1 [79.9–147.6]	40.0 [29.3–47.8]	*p* < 0.0001^d^
Aβ42 (pg/ml)	511 [421–623]	420 [372–575]	1240 [1036–1600]	*p* < 0.0001^d^
NLK (ng/ml)	3.88 [3.00–5.24]	4.17 [3.61–4.60]	2.82 [2.30–3.60]	*p* < 0.0001^d^
YKL-40 (ng/ml)	*n* = 13; 324 ± 63	*n* = 5; 323 ± 40	*n* = 29; 226 ± 51	*p* < 0.0001^a^

Characteristics of the Barcelona AD-control group. Medians and [IQR] are reported, except for age and YKL-40 (mean ± SD), and sex (*n*). TP, T-tau, P-tau, Aβ42, NLK and YKL-40 were measured in CSF

*Abbreviations: AD* Alzheimer’s disease, *Aβ42* amyloid beta-42, *APOE* Apolipoprotein E, *aMCI* amnestic mild cognitive impairment, *F* female, *IQR* interquartile range, *M* male, *MMSE* Mini-Mental State Examination, *N.A.* not available, *NLK* neuroleukin, *P-tau* phosphorylated tau, *SD* standard deviation, *TP* total protein, *T-tau* total tau, *YKL-40* protein associated with neuro-inflammation and Alzheimer’s disease, named after the first three N-terminal amino acids: tyrosine (Y), lysine (K), and leucine (L) and its molecule mass (40 kDa), also known as chitinase-3 like-protein 1

^a^ANOVA

^b^Chi-square test

^c^Fisher’s exact test

^d^Kruskal-Wallis test

We found a higher mean CSF NLK (4.44 ± 1.28 ng/ml) in *n* = 40 aMCI/AD patients with positive AD biomarkers (A+T+(N+)) than in *n* = 41 controls with normal AD biomarker status (A−T−(N−), 3.15 ± 0.91 ng/ml, *p* = 0.001; Fig. 1E), when adjusted for age and sex. This difference remained after additional adjustment for total protein (*p* = 0.001).

Again, NLK correlated with age (*r*_SP_ = 0.43, *p* < 0.0001) and with total protein (*r*_SP_ = 0.32, *p* < 0.0001).

### ROC analysis

**Fig. 2:**
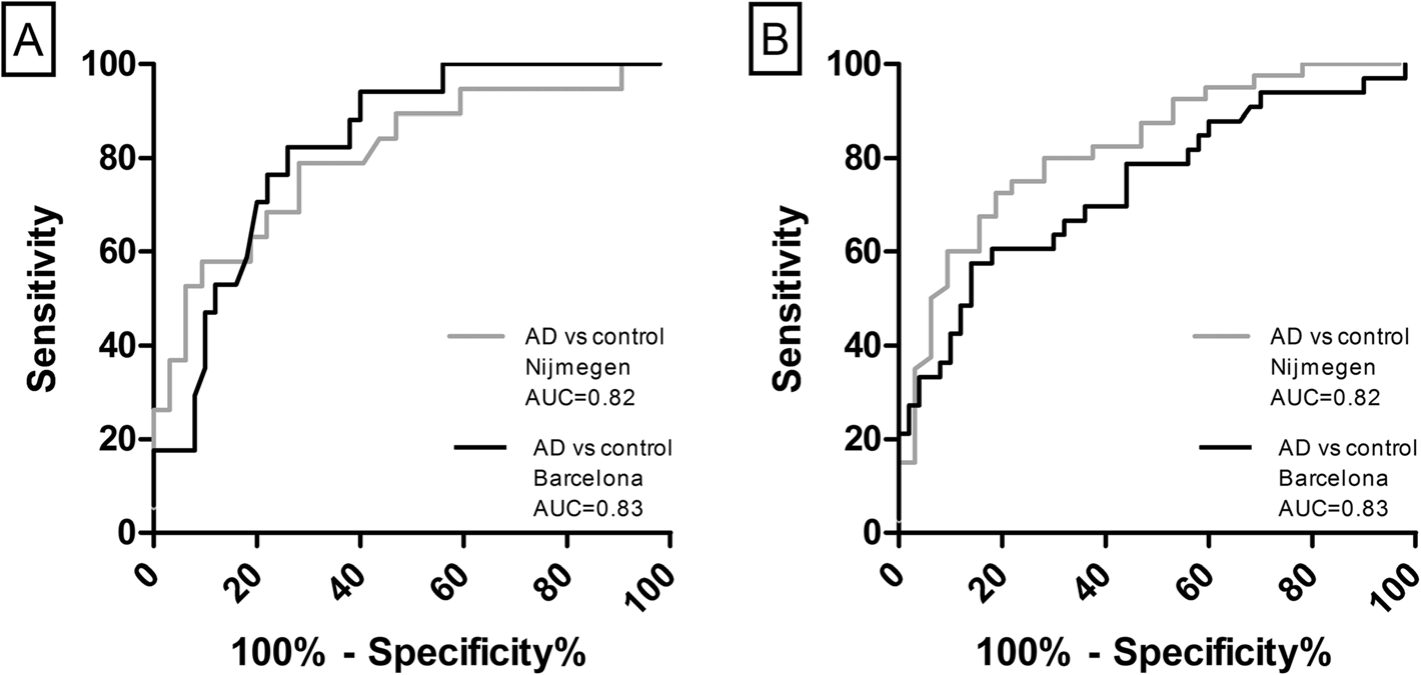
**A** ROC analysis showed moderately high accuracy levels for discrimination of aMCI from control in the Nijmegen aMCI patients and controls (gray line) and the Barcelona aMCI patients and controls. **B** ROC analysis showed consistently high accuracy levels for discrimination of AD from control in the Nijmegen AD patients and controls (gray line) and the Barcelona AD patients and controls. Abbreviations: AD, Alzheimer’s disease; AUC area under the curve. The Barcelona cohort serves as a validation cohort

We found an AUC of 0.81 (95% CI 0.68–0.93; Fig. 2A) to discriminate aMCI patients from controls in the Nijmegen sample, and an AUC of 0.74 (95% CI 0.63–0.85) in the Barcelona sample.

We found an AUC of 0.82 (95% CI 0.73–0.92; Fig. 2B) to discriminate AD patients from controls in the Nijmegen sample, and an AUC of 0.83 (95% CI 0.73–0.93) in the Barcelona sample.

### Correlation of CSF NLK with other CSF biomarkers and MRI markers

**Fig. 3:**
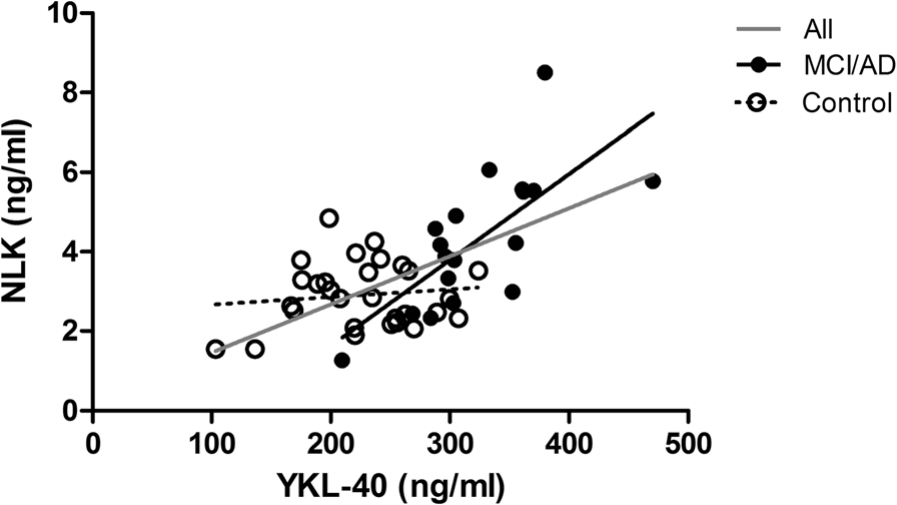
Scatter plot of the correlation between CSF NLK levels and YKL-40 levels in a subset (*n* = 47) of the Barcelona aMCI/AD patients and controls. NLK and YKL-40-levels significantly correlated in the combined aMCI/AD and controls (*r*_SP_ = 0.51, *p* < 0.0001, gray line) and in the aMCI/AD patients separately (*r*_SP_ = 0.79, *p* < 0.0001, black dots and black line), but not in the controls (*r*_SP_ = 0.033, *p* = 0.87, white dots, dotted line). Abbreviations: AD, Alzheimer’s dementia; r_SP_, Spearman rank coefficient; aMCI, amnestic mild cognitive impairment; NLK, neuroleukin; YKL-40, chitinase-3 like-protein 1

CSF NLK correlated with CSF YKL-40 (Spearman rank coefficient, adjusted for age (*r*_SPc_) = 0.34, *p* < 0.019) in the combined Barcelona aMCI/AD patients and controls (Fig. 3). This correlation was prominent in the aMCI/AD patients (*r*_SPc_ = 0.82, *p* < 0.0001), but not in the controls (*r*_SPc_ = −0.14, *p* = 0.47).

CSF NLK levels correlated with CSF t-tau levels (Nijmegen aMCI/AD patients and controls: *r*_SPc_ = 0.65, *p* < 0.0001; Barcelona aMCI/AD patients and controls *r*_SPc_ = 0.60, *p* < 0.0001) and CSF p-tau levels (Nijmegen aMCI/AD patients and controls: *r*_SPc_ = 0.65, *p* < 0.0001; Barcelona aMCI/AD patients and controls: *r*_SPc_ = 0.58, *p* < 0.0001). CSF NLK correlated with Aβ42 levels in the Nijmegen aMCI/AD patients and controls (*r*_SPc_ = −0.29, *p* = 0.005), but not in the Barcelona aMCI/AD patients and controls (*r*_SPc_ = −0.014, *p* = 0.89).

There was neither a correlation between CSF NLK and GCA score (*r*_SPc_ = 0.23, *p* = 0.17), nor with mean MTA score (*r*_SPc_ = −0.036, *p* = 0.84).

## Discussion

The main findings of our study are as follows: (1) CSF NLK levels were increased in aMCI patients and in AD patients, as established in two independent samples from two different centers. (2) The increase in CSF NLK was particularly strong in “AD biomarker positive A+T+(N+)” aMCI and AD patients compared to A−T−(N−) controls. (3) CSF NLK levels were associated with CSF YKL-40 levels in aMCI/AD patients, which suggests an association of NLK with neuroinflammation. (4) CSF NLK levels were not increased in sporadic CAA patients versus controls.

NLK is a 56-kDa multifunctional protein named after two of its functions: it can act as a neurotrophic factor and as a lymphokine [12]. In the extracellular space NLK supports neuronal survival and stimulates axonal growth [10–13], but NLK may also play a role in the induction of B-cell differentiation into antibody-secreting cells [9]. NLK is also known as glucose-6-phosphate isomerase or phosphohexose isomerase, an intracellular enzyme involved in glycolysis and gluconeogenesis [31], as autocrine motility factor, a tumor maturation factor in cancer cells, playing a role in metastasis [32] and as a maturation factor, mediating differentiation of human myeloid leukemia cells [33]. In an immunohistochemical study in AD patients, NLK expression was found in neurons, microglia, and oligodendroglia, and in the vessel wall, specifically in cerebrovascular cells such as smooth muscle cells [8]. NLK has also been investigated in the context of other neurodegenerative diseases: in a transgenic mouse model of Huntington’s disease, the NLK gene was upregulated [34]. Furthermore, in patients with Parkinson’s disease and multiple system atrophy, CSF NLK levels were normal [25].

Our finding that CSF NLK is increased in aMCI patients suggests that altered levels are already present in prodromal AD, and that NLK might serve as an early biomarker for AD. Levels were also altered in AD patients. We found similar effects sizes and AUC’s of CSF NLK in aMCI patients and AD patients in two independent samples of patients and controls, indicating that our results are consistent. However, there is overlap of NLK levels between aMCI patients and controls. More studies will be needed to confirm this association of NLK with different stages of AD as well as its potential biomarker value for early diagnosis.

We observed a marked increase of NLK in aMCI/AD with a positive AD biomarker status, and a clear correlation with CSF p-tau and t-tau, whereas we found no or only a weak correlation of NLK with CSF Aβ42. Together, these findings suggest that an increased cerebral NLK production is tightly associated with tau-mediated neurodegeneration.

In line with this, we found that CSF NLK strongly correlated with CSF YKL-40 (also known as chitinase-3 like-protein 1 or cartilage glycoprotein-39), an established neuroinflammatory marker [14], expressed by astrocytes and microglia. Furthermore, our finding that this association is very strong in the aMCI/AD group suggests that a neuroinflammatory process involving increased production of NLK and YKL-40 may be particularly associated with the early phases of the disease process.

Chronic microglial inflammation has been observed in the brains of AD patients at various disease stages, and pro-inflammatory cytokines such as IL-1β, IL-2, IL-6, and tumor necrosis factor-α have been shown to be upregulated in AD [35]. In addition, genome-wide association studies have identified associations between inflammation- and immune-related variants in genes (such as a missense mutation in the gene encoding for TREM2, a receptor expressed on microglia) and increased AD risk [36]. Furthermore, Aβ may initiate microglial activation, resulting in microglial migration to plaques, phagocytosis, and enzymatic degradation of Aβ [37]. Prolonged microglial activation may be detrimental, since it eventually leads to a self-perpetuating cycle involving the production of neurotoxic pro-inflammatory cytokines, damaging neurons, inducing more recruitment of microglia, further increasing their activation and release of pro-inflammatory cytokines [35].

Inflammation does, however, not seem to be a major component in CAA-related microhemorrhage or microinfarction [38], with the exception of a rare clinical subtype: CAA-related inflammation [6]. On the other hand, previous immunohistochemical studies in AD patients and in a transgenic rat model of CAA have found that CAA type 1, i.e., amyloid deposition in the capillary vessels, was associated with perivascular inflammation [39, 40].

We previously demonstrated by immunohistochemistry that NLK may be associated with CAA pathology [8], but apparently this association is not reflected by an increase of NLK in the CSF. We could speculate that CAA pathology, in contrast to AD pathology, does not lead to a neuroprotective response involving NLK to an extent in which that is reflected by an increase of NLK in the CSF. The correlation between NLK and MMSE levels might suggest that NLK levels in CAA patients are influenced by cognition (and possibly additional AD pathology). However, since we only have the availability of MMSE scores in a subset of CAA patients, we should interpret these data with caution.

### Strengths and limitations

A limitation of this study is that we did not have YKL-40 levels available for the Nijmegen CAA, AD patients, and controls. However, the correlation in the Barcelona samples is quite strong. In addition, the lack of detailed information on the cognitive status of CAA patients obstructed us from systematically analyzing this as a possible confounding factor for the analysis of NLK levels. Furthermore, controls were significantly younger than the aMCI patients and AD patients. However, when we adjusted for age, difference between patients and controls remained significant. Our group sizes were relative small, but a strength of our study is the inclusion of aMCI/AD and controls subjects with thorough clinical evaluation, for many of whom imaging and AD biomarker data was also available. Another important strength is that we were able to validate our findings in two independent aMCI/AD samples.

## Conclusions

We conclude that CSF NLK is increased in aMCI and AD patients, but not in CAA patients, which indicates that it is a marker associated with early stage AD. NLK is a neurotrophic factor, possibly involved in a neuroprotective response to neuronal damage caused by AD pathology. The strong correlation of NLK with YKL-40 suggests an inflammatory role for NLK as well. Future studies will have to confirm whether an increase in CSF NLK is associated with early stages of AD and whether NLK has an additive value as biomarker for the differential diagnosis of dementia syndromes.

## Data Availability

The datasets used and/or analyzed during the current study are available from the corresponding author upon reasonable request.
