# Correction to: Cerebrospinal fluid levels of the neurotrophic factor neuroleukin are increased in early Alzheimer’s disease, but not in cerebral amyloid angiopathy

**DOI:** 10.1186/s13195-021-00929-x

**Published:** 2021-11-16

**Authors:** Anna M. De Kort, H. Bea Kuiperij, Daniel Alcolea, Iris Kersten, Alexandra A. M. Versleijen, Steven M. Greenberg, Erik Stoops, Floris H. B. M. Schreuder, Catharina J. M. Klijn, Alberto Lleó, Jurgen A. H. R. Claassen, Marcel M. Verbeek

**Affiliations:** 1 Department of Neurology, Radboud University Medical Center, Donders Institute for Brain, Cognition and Behaviour, Radboud Alzheimer Centre, P.O. Box 9101, 6500 HB Nijmegen, The Netherlands; 2 Sant Pau Memory Unit, Department of Neurology, Hospital de la Santa Creu i Sant Pau, Biomedical Research Institute Sant Pau, Universitat Autònoma de Barcelona, Barcelona, Spain; 3 Center of Biomedical Investigation Network for Neurodegenerative Diseases (CIBERNED), Madrid, Spain; 4 Department of Laboratory Medicine, Radboud University Medical Center, Nijmegen, The Netherlands; 5 Department of Neurology, Massachusetts General Hospital, Harvard Medical School, Boston, MA USA; 6 ADx NeuroSciences, Ghent, Belgium; 7 Department of Geriatrics, Radboud University Medical Center, Donders Institute for Brain, Cognition and Behaviour, Radboud Alzheimer Centre, Nijmegen, The Netherlands

**Correction to: Alz Res Therapy 13, 160 (2021)**

**https://doi.org/10.1186/s13195-021-00899-0**

Following the publication of the original article [1] the authors noticed that the published Fig. 2 is incorrect. The authors uploaded the incorrect figure during the proofing process. The original article [1] has been updated. The correct Fig. 2 is depicted below.

**Fig. 2:**
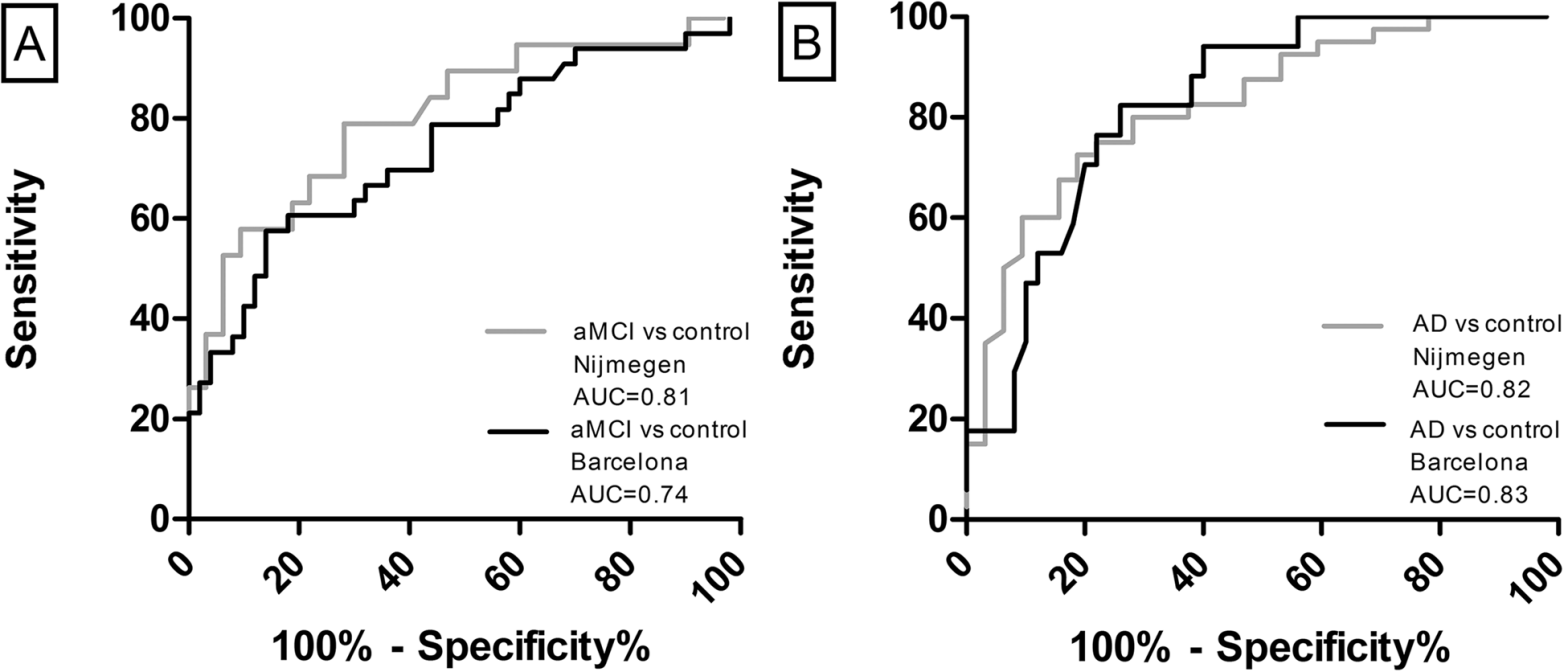
**A** ROC analysis showed moderately high accuracy levels for discrimination of aMCI from control in the Nijmegen aMCI patients and controls (gray line) and the Barcelona aMCI patients and controls (black line). **B** ROC analysis showed consistently high accuracy levels for discrimination of AD from control in the Nijmegen AD patients and controls (gray line) and the Barcelona AD patients and controls (black line). Abbreviations: AD, Alzheimer’s disease; AUC area under the curve. The Barcelona cohort serves as a validation cohort
